# Posterior choroidal boundary morphology and segmentation errors influence on choroidal thickness assessment in diabetic patients – a swept-source OCT study

**DOI:** 10.22336/rjo.2021.45

**Published:** 2021

**Authors:** Otilia Obadă, Anca Delia Pantalon, Gabriela Rusu-Zota, Anca Hăisan, Ioana Smaranda Lupuşoru, Daniel George Boicu, Dorin Chiseliţă

**Affiliations:** * Department of Ophthalmology, “Gr. T. Popa” University of Medicine and Pharmacy, Iaşi, Romania; ** Department of Ophthalmology, “Sfântul Spiridon” Clinical Emergency Hospital, Iaşi, Romania; *** Department of Pharmacology, “Gr. T. Popa” University of Medicine and Pharmacy, Iaşi, Romania; **** Department of Emergency Medicine, “Gr. T. Popa” University of Medicine and Pharmacy, Iaşi, Romania; ***** Department of Surgery, “Sfântul Spiridon” Clinical Emergency Hospital, Iaşi, Romania; ****** “Gr. T. Popa” University of Medicine and Pharmacy, Iaşi, Romania; ******* “Oftaprof” Eye Clinic, Iaşi, Romania

**Keywords:** choroidal segmentation, choroidoscleral junction, segmentation method, choroidal morphology, diabetes mellitus

## Abstract

**Objective:** to evaluate the choroidal morphology and choroidal thickness (CT) in normal and diabetic subjects and to compare the differences between automated segmentation (AS) and manual segmentation (MS) of the choroid.

**Methods:** in this observational cross-sectional study we included 48 eyes: 24 normal eyes (group 1), 9 eyes with DM without diabetic retinopathy (DR) (group 2) and 15 eyes with DM and DR (group 3). Swept-source OCT line scans images were analyzed for the presence of the suprachoroidal layer (SCL), choroidal morphology and the CT was measured manually subfoveal and at 750 μ both nasal and temporal to the fovea after AS and MS. SCL was not included in the CT evaluation. CT values were compared between the groups and between the three points of evaluation.

**Results:** SCL was visualized in 21 eyes (43.8%). In diabetic patients, SCL was visible in 11 (45.83%) cases and in nondiabetic patients, in 10 eyes (41.66%). There was a good AS of Bruch’s membrane, which was not further corrected manually. There were statistically significant differences between AS and MS at the level of CSJ for all three locations in all three groups (P ≤ 0.01). After MS, the choroid was statistically significantly thicker. Group 2 and group 3 showed a higher CT thickness. There were no statistically significant differences in the CT between groups in all three locations.

**Conclusions:** Defining posterior choroidal boundary and the applied segmentation method can result in differences in CT measurements. Diabetic patients have altered CT and choroidal morphology.

**Abbreviations:** CT = choroidal thickness, AS = automated segmentation, MS = manual segmentation, CSJ = choroidoscleral junction, SCL = suprachoroidal layer, SCS = suprachoroidal space, DM = diabetes mellitus, DR = diabetic retinopathy, RPE = retinal pigmented epithelium, BM = Buch’s membrane

## Introduction

The choroid is a complex highly vascular tissue with an important role in nourishing the retinal pigmented epithelium (RPE) and the outer retina and accounts for most of the ocular blood flow [**1**]. The choroid is composed of a three vascular layer (large and medium vessels and the choriocapillaris layer) and a stroma consisting of pigmented cells (giant melanocytes) and nonpigmented cells (fibroblasts), collagen and elastic fibers [**2**]. The limits of the choroid are represented by the lamina vitrea or Buch’s membrane (BM) anteriorly and lamina fusca or suprachoroidal layer (SCL) posteriorly [**3**]. If the anterior border of the choroid is a compact one, the posterior border is a transition zone with variable thickness [**4**], approximatively 30 μ. 

The suprachoroidal space (SCS) is the virtual space between the choroid and the sclera in the healthy eyes, which becomes physically apparent when the lamellae of the SCL separate in pathological conditions [**2**,**4**].

The posterior boundary segmentation of the choroid is the key to automated segmentation (AS). The choroidoscleral junction (CSJ) or interface is a transition zone where the choroid meets the sclera [**5**]. An important factor that influences the CT measurements is the definition of the CSJ [**3**,**6**]. The proposed definition of the CSJ is a line that separates the choroidal vascular layer from the sclera rather than a separate layer [**2**]. 

OCT structure of the choroid can be analyzed both on spectral domain OCT (SD-OCT) and swept-source OCT (SS-OCT). Due to the longer central wavelength of the SS-OCT (1060 nm vs. 840 nm in SD-OCT), there is a deeper penetration through RPE and the CSJ has a higher contrast and therefore a better visualization [**2**,**7**,**8**]. CSJ was described on OCT either as a single distinct junction when the SCL is not visible (SCL absent), either as a wide hyporeflective band representing the SCL (SCL present) [**3**,**6**]. The SCL is defined as a more hyporeflective band compared to the choroidal stroma [**6**,**9**].

Automated choroidal segmentation is achieved by means of a built-in software and averaging multiple frames of images increase layer detection and enhance image quality by speckle suppression and signal-to-noise ratio improvement [**10**].

## Methods

This is a prospective observational cross-sectional study performed in the Ophthalmology Department of “Sfântul Spiridon” Clinical Emergency Hospital of Iași, Romania, between August and January 2017. The study was conducted in accordance with the Declaration of Helsinki and the written consent was obtained from all patients according to the protocol approved by the Ethics Committee of “Gr. T. Popa” University of Medicine and Pharmacy, Iași. All patients underwent a comprehensive ophthalmic examination including best corrected visual acuity (BCVA), Goldmann applanation tonometry, slit-lamp examination, fundus examination after mydriasis with Tropicamide 1%, axial length (AL) with contact ultrasound ocular biometry (ALCON Ultra Scan Imaging System) and swept-source ocular coherence tomography (SS-OCT) with mydriasis. Fasting blood glucose and glycated haemoglobin (HbA1c) were evaluated. 

Our study included Caucasian subjects with type 1 or type 2 diabetes mellitus (DM) and nondiabetic patients. The individuals were divided into 3 groups: group 1 (nondiabetic patients), group 2 (diabetic patients without diabetic retinopathy, DR) and group 3 (diabetic patients with DR). We excluded the subjects under 18 years old, with BCVA > 1 LogMAR, with refractive errors over -6 spherical diopters, +4 spherical diopters and ±5 cylindrical diopters. The subjects who underwent vitreoretinal surgery, intravitreal injections, and those with ocular disease (glaucoma, ocular hypertension, uveitis, maculopathies others than those associated with DM) and those with conditions interfering with good quality image acquisition (dense cataract, vitreous haemorrhage, corneal leucoma) were also excluded. Our study did not include patients with DM and diabetic macular oedema (DME).

**Fig. 1:**
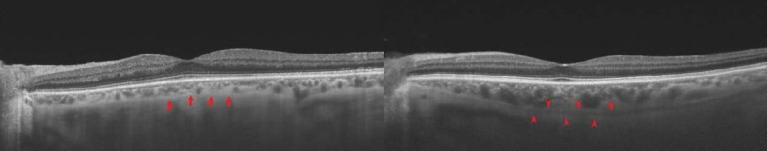
The suprachoroidal layer (SCL) absent (left, red arrows), SCL present (right, arrowheads)

**Fig. 2:**
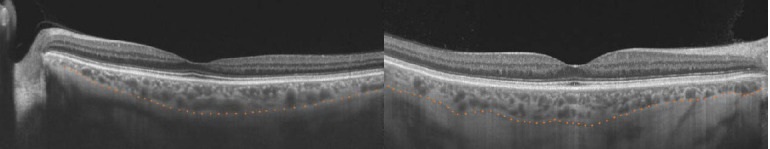
Choroidal shape and contour (orange dots): regular choroid with convex contour (left); irregular choroid with concave-convex-concave contour (right)

**Fig. 3:**
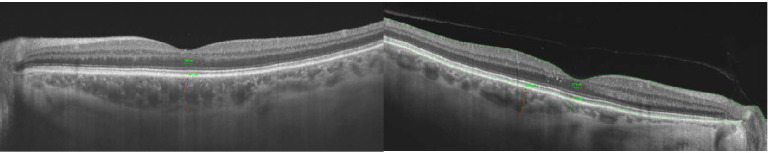
Thickest choroidal point (red dotted line): subfoveal (left); not subfoveal (right)

OCT images were obtained with SS-OCT (DRI Triton; Topcon Corporation, Tokyo, Japan) with a built-in automated choroidal segmentation software. For each subject, we performed two types of OCT scans after mydriasis: a macular cube scan of 7.0 x 7.0 (512 x 128) to evaluate the structure of the macula and a 9 mm 1024-line scan centered on the fovea for choroid assessment. For each image, EVV (Enhanced Vitreous Visualization) was set off, and the Y scale was set at 1:2. Each image was analyzed for the presence of SCL and the patients were classified as having SCL present or SCL absent (**Fig. 1**). The choroidal morphological features analyzed were the choroidal contour regularity and the position of the thickest choroidal point. A regular choroid shape was defined as a choroid with a convex contour and an irregular choroid shape was defined as a choroid with concave-convex-concave contour (**Fig. 2**). The thickest choroidal point was subfoveal when it was located within 100 μ nasal and 100 μ temporal to the fovea (**Fig. 3**) [**11**].

**Fig. 4:**
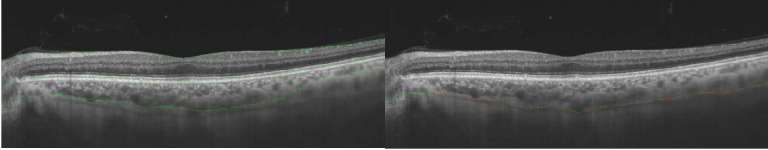
SS-OCT line scan: AS of the CSJ (left); CSJ after manual correction (right)

The choroid was segmented automatically (automated segmentation–AS) between the outer border of the hyperreflective band corresponding to the RPE–BM complex and CSJ. Using IMAGEnet software, the CT was measured manually perpendicularly between the hyperreflective line of the BM and the CSJ in three points: subfoveal (SF) and at 750 μ both nasal (750 N) and temporal (750 T) from the fovea. SCL was not included in the assessment of the CT. Each image was analyzed for the presence of errors in the anterior and posterior segmentation line, which were then corrected manually (manual segmentation-MS) (**Fig. 4**). Then, the choroid was manually measured between the new segmentation lines. The values of the CT were statistically analyzed for differences between the two methods of segmentation in the three groups. 

**Statistical analysis**

Data was analyzed using SPSS version 26.0 (IBM Corp., Armonk, NY, USA) and Excel, Microsoft Office. Shapiro-Wilk test was used to test the normality of data distribution. One-way ANOVA test was used for normal distributions when comparing the variables between groups and Kruskal-Wallis test for non-normally distributed variables. The paired sample t-test was used to compare the means of the AS vs. MS measurements. Fisher᾿s exact test was used for analyzing the categorical data. Quantitative data for AS and MS measurements were reported as minimum, maximum, mean, standard deviation (SD). A P value < 0.05 was considered statistically significant. 

## Results

Our study included 48 patients (48 eyes): 24 nondiabetic patients (24 eyes) in group 1 and 24 diabetic patients (24 eyes): 9 without DR (group 2) and 15 with DR (group 3). The mean age for nondiabetic patients (group 1) was 71.29 ± 9.35 years (50–86); the mean age for diabetic patients (group 2 and group 3) was 67 ± 8.59 (49–82), with no statistically significant differences. There were no statistically significant differences between the three groups with respect to mean BCVA, SE, IOP and axial length (**Table 1**).

**Tabel 1:** Ocular and systemic characteristics

	**Group 1** (n=24)	**Group 2** (n=9)	**Group 3** (n=15)	***P*** value
**Age** (years)				
mean ± SD	71.29 ± 9.35	71.78 ± 9.48	64.13 ± 6.81	0.034*
variation	50–86	58–82	49–74	
**Gender**				
(female:male)	13:11	6:3	11:4	0.53†
**BCVA** (logMAR)				
mean ± SD	0.22 ± 0.22	0.26 ± 0.11	0.21 ± 0.26	0.36‡
variation	0–1	0.1–0.4	0–1	
**IOP** (mmHg)				
mean ± SD	13.88 ± 3.20	14.67 ± 2.82	16.27 ± 2.76	*0.06‡*
variation	9–20	10–18	11–21	
**SE** (D)				
mean ± SD	0.06 ± 1.37	-0.68 ± 1.67	0.53 ± 1.56	*0.06‡*
variation	-1.25–4	-4.12–1.75	-3.5–2.75	
**Lens status**, *n* (%)				
- phakic	15 (62.50)	7 (77.77)	13 (86.66)	–
- pseudophakic	9 (37.50)	2 (22.23)	2 (13.33)	
**Axial length** (mm)				
mean ± SD	22.93 ± 0.78	22.87 ± 0.66	22.82 ± 0.64	0.89*
variation	21.41–24.61	22.10–24.26	21.73–24	
**Fasting blood glucose** (mmol/l) §				
mean ± SD	5.50 ± 0.73	7.18 ± 1.66	10.64 ± 4.59	0.00*
variation	3.55–6.72	5.44–10.66	4–20.81	
**HbA1c** (%)				
mean ± SD	5.56 ± 0.40	7.80 ± 3.11	8.09 ± 2.11	*0.00‡*
variation	4.93–6.60	5.50–15.50	5.31–12.02	
**DM duration**||				
mean ± SD	–	4.41 ± 3.70	11.40 ± 7.55	*0.01#*
variation	–	0.16–10	4–31	
**DM treatment**, *n* (%)				
- OAD	–	8 (88.88)	7 (46.66)	–
- OAD + insulin	–	1 (11.11)	8 (53.33)	
**Controlled high BP**, *n* (%)	22 (91.66)	8 (88.88)	12 (80)	–
SD = standard deviation; ODA = oral antidiabetics; D = diopter; BP = blood pressure ; *one-way ANOVA test for normal distributed data; all comparison were corrected with post hoc test ; † Fisher՚s exact test ; ‡ Kruskal-Wallis test for nonnormal distributed data; all comparisons were corrected with post hoc test ; § mmol/ l x 18,0182 = mg/ dl ; || years of positive DM diagnosis ; # Mann-Whitney U test				

Average quality index (QI) was 97.70 (84–100) and average for averaging success rate was 120.08 (62-128).

**Fig. 5:**
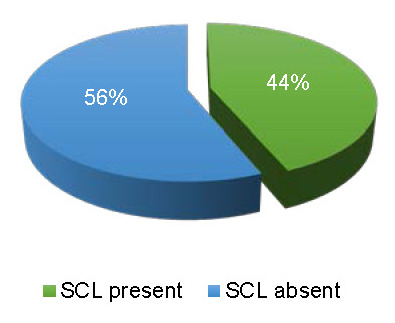
SCL distribution for the analyzed images

SCL was visualized in 21 eyes (43.8%) (**Fig. 5**), from which only 13 eyes (61.90%) had a complete hyporeflective band; in 6 eyes (28.57%), SCL was present only temporal to the fovea. In diabetic patients, SCL was visible in 11 eyes (45.83%) and in nondiabetic patients, SCL was visible in 10 eyes (41.66%). 

Choroidal morphological features in the three study groups are presented in **Table 2**. There was a good AS of BM, which was not further corrected manually. There were statistically significant differences between AS and MS at the level of CSJ for all three locations in all three groups (P ≤ 0.01); the choroid was statistically significantly thicker after MS in all three locations (**Table 3**).

**Tabel 2:** Choroidal morphological features in the study groups

**Morphologic feature**	**Group 1** (n=24)	**Group 2** (n=9)	**Group 3** (n=15)
**Irregular choroid**	41.66% (10)	77.77% (7)	80% (12)
**Thickest choroidal point not subfoveal**	29.16% (7)	33.33% (3)	73.33% (11)

**Tabel 3:** Choroidal thickness among group

**CT (µ)**	**Group 1** (n=24)	**Group 2** (n=9)	**Group 3** (n=15)	***P*** Value
**SF**				
***AS***				
mean ± SD	205.38 ± 110.03	210.56 ± 86.69	205.80 ± 86.21	1.00*
variation	35-426	86-358	66-330	
***MS***				
mean ± SD	242.50 ± 114.43	244.33 ± 96.56	280.67 ± 87.66	0.51*
variation	77-449	106-403	138-465	
***P*** *Value*	0.004†	0.001†	0.005†	
**750 T**				
***AS***				
mean ± SD	204.88 ± 91.68	213.78 ± 64.55	215.87 ± 82.29	0.91*
variation	32-364	128-303	53-318	
***MS***				
mean ± SD	246.08 ± 94.13	249.89 ± 85.28	282.40 ± 76.88	0.43*
variation	104-420	148-391	141-416	
***P*** *Value*	0.000†	0.007†	0.014†	
**750 N**				
***AS***				
mean ± SD	183.50 ± 114.62	202.89 ± 83.58	218.60 ± 88.10	0.57*
variation	31-441	56-311	58-366	
***MS***				
mean ± SD	227.17 ± 121.95	235.33 ± 94.54	285.67 ± 99.38	0.27*
variation	61-504	68-334	152-482	
***P*** *Value*	0.002†	0.002†	0.003†	
*one-way ANOVA test for normal distributed data; all comparison were corrected with post hoc test ; † paired sample t-test				

**Fig. 6:**
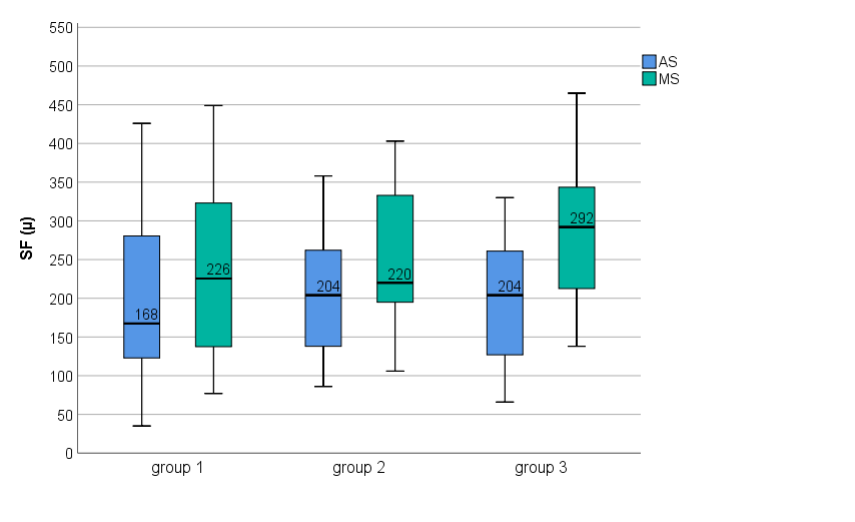
SF choroidal thickness among three groups

**Fig. 7:**
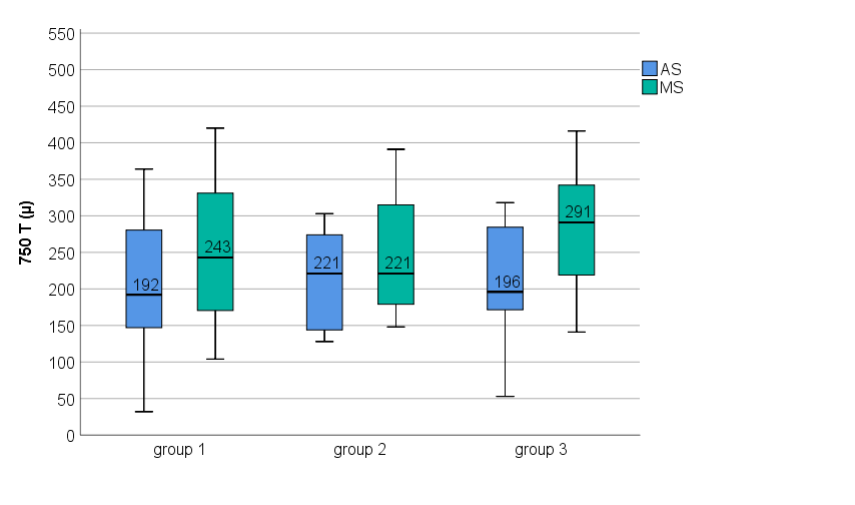
750 T choroidal thickness among three groups

**Fig. 8:**
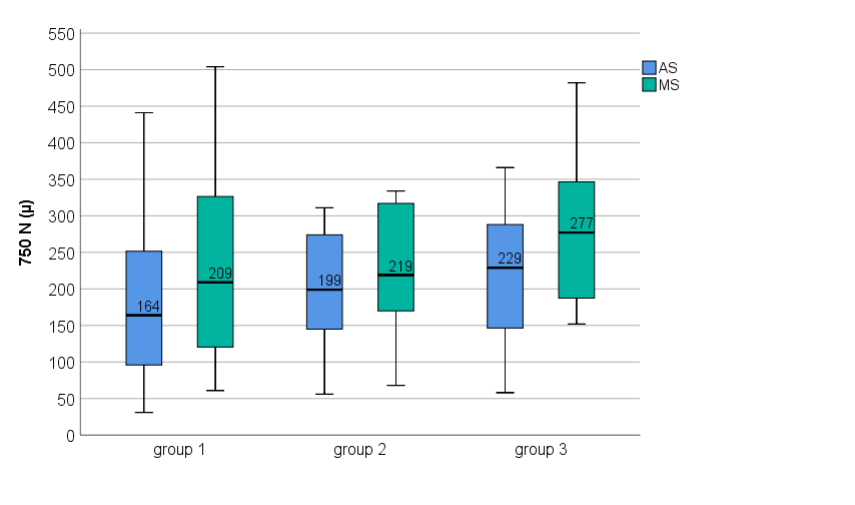
750 N choroidal thickness among three groups

CT measured at 750 T point and 750 N point was higher in group 2 and group 3, compared with group 1, but with no statistically significant differences were observed. There were no statistically significant differences in the CT between groups in all three locations, but P values were lower after MS (**Table 3**, **Fig. 6**-**8**). 

**Fig. 9:**
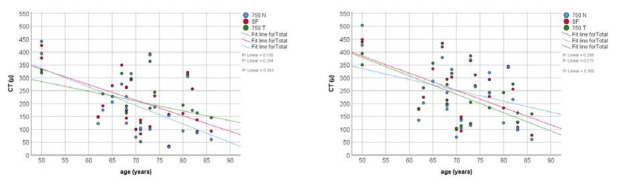
Correlation between CT and age for AS (left) and MS (right) measurements in group 1

There was a statistically significant negative correlation of CT with age for the patients in group 1 for SF choroid (r=-0.513, P=0.01 for AS, and r=-0.546, P=0.006, for MS respectively), 750 T choroid (r=-0.387, P=0.05 for AS, and r=-0.418, P=0.042, for MS respectively), and 750 N choroid (r=-0.586, P=0.003 for AS, and r=-0.548, P=0.006, for MS respectively) (**Fig. 9**) and no correlation for the patients in group 2 and 3. There was no correlation between CT and axial length in all three groups.

## Discussion

CT varies largely in different studies, depending on various factors. In our study, we emphasized that the posterior segmentation line is an important factor that can modify the CT. Also, we evaluated the CT after AS and MS of the posterior choroidal border. Kong et al. reported a high error rate for automated choroidal segmentation in eyes with choroidal alteration that could not be reduced by the averaging effect. Also, they identified automated choroidal segmentation errors more often at the posterior border than at the anterior one [**10**]. 

In our study, we found a thicker choroid after MS, although we did not include the SCL in the measurements. Most commonly, the AS line did not reach the CSJ, but stopped at the posterior large-vessel border of the vascular choroidal layer, minimizing the contribution of the stromal choroidal layer to the total choroidal thickness.

SS-OCT allows the exact identification of the CSJ. In our study, we could delineate correctly the posterior choroidal boundary in all the investigated patients. In a SS-OCT study on healthy eyes and eyes with different retinal diseases, Michalewska et al. observed the outer choroidal boundary in all cases [**12**]. 

The SCL is not present or visible in every subject. Gupta et al. studied the CSJ in Asian eyes with SD-OCT and could not appreciate the presence of SCL [**6**], possibly due to a greater pigmentation in these eyes compared to Caucasian eyes. Studies revealed that 45% subjects have a SCL visible on OCT images [**3**]. However, even when SCL is present, it is not always present throughout the entire OCT line scan. In our study, SCL was present in 21 eyes (43.8%), from which only 13 eyes (61.90%) had a complete hyporeflective band; in 6 eyes (28.57%) SCL was present only temporal to the fovea.

In their SD-OCT study about the influence of ocular and systemic factors to the visibility of the choroidoscleral interface, Gupta et al. reported a 60% well-defined CSJ in healthy eyes and identified younger age, shorter axial length, thicker retina, and diabetes mellitus as factors affecting the visibility of the CSJ and CT measurements [**6**]. The poorer CSJ visibility in patients with DM was speculated to be associated with changes in choroidal blood flow, shadowing effect of the choroid due to the intraretinal fluid and cataract [**6**,**11**]. 

Adhi et al. analyzed the morphological features of the choroid in a SD-OCT study on patients with NPDR without DME, PDR without DME and DME compared with healthy subjects and identified that the irregular choroidoscleral interface contour, displacement of the thickest point of the choroid from under the fovea and focal choroidal thinning are more frequent in patients with DR with or without DME [**11**]. In our study, we analyzed the choroidal contour and the displacement of the thickest point of the choroid from under the fovea in healthy subjects and in patients with DM without DR and with DR and found that over 77% diabetic patients had an irregular choroidal contour. We also observed that 41.66% patients with no DM presented with an irregular choroidal contour. On the other hand, Adhi et al. and Branchini et al. reported a 100% regular, bowl-shaped choroidal contour in healthy subjects [**11**,**13**]. When analyzing the horizontal macular choroidal profile in a SS-OCT study, Ruiz-Medrano et al. found that 87.2% healthy eyes had a regular, bowl-shaped choroidal contour and 12,8% had a temporal choroidoscleral interface inflection attributed to the insertion of the inferior oblique muscle [**14**]. Gupta et al. analyzed the choroidal morphological features in Indian adults with diabetes and observed a more irregular contour of the CSI with an increase in the number of the inflection points, suggesting that there is an association between these and focal choroidal changes. When comparing between DM without DR and DM with DR, Gupta et al. found fewer inflection points with regularization of the CSI contour as the expression of a possible lesser local variation in choroidal vascularization [**15**]. Ruiz-Medrano’s study included individuals with no systemic or ocular diseases, except for cataract. In our study, there was a high proportion (41.66%) of nondiabetic patients with irregular choroidal contour. This can be related to the fact that the majority had controlled high blood pressure, which, on a long stand course, could induce alteration in choroid function and morphology. 

With respect to the location of the thickest choroidal point, the results in our study were consistent with those in other studies [**11**,**13**]. Adhi identified 96% and Branchini 88% of healthy eyes having the thickest choroidal point under the fovea. In our study, 70.84% eyes had the thickest of choroidal point under the fovea; displacement of this point from under the fovea was present in 33.33% patients with DM and 73.33% patients with DM with DR. 

Reports on CT measurements in DM and DR presented conflicting results, with no clear-cut consensus [**9**]. In a SS-OCT study, Wang et al. reported a higher CT in the early stage of DR, which further decreased with DR progression [**16**]. They proposed that DM could be an independent factor contributing to choroidal thickening. Afterwards, the progression of DR could lead to a thinning of the choroid. Also, Lains et al. reported a significant reduction of CT only in patients with proliferative DR compared with controls [**17**]. Kim et al. reported a significantly higher CT as severity of DR worsened from mild DR to proliferative DR. CT decreased in eyes treated with panretinal photocoagulation [**18**].

In the population-based Beijing Eye Study, Xu et al. evaluated the subfoveal CT in patients with DM and patients with DR and concluded that the first had a slightly, but statistically significant, thicker subfoveal choroid. Moreover, DR and the stage of DR were not associated with abnormal subfoveal CT [**19**]. 

In our study, there were no significant differences between the three groups for the subfoveal CT. We identified a thicker choroid in patients with DM and those with DM and DR at 750 μ nasal and 750 μ temporal from the fovea, but with no significant differences. These findings could be due the small sample size or because of discrete choroidal changes.

In a PubMed database systematic review of the recent literature about choroidal structural alteration in diabetic patients, Hamadneh et al. gathered and classified the analyzed studies in different categories related to increasing, decreasing or no change in CT in DM patients or the progression of DR and concluded that CT is an unreliable parameter because it has a poor correlation with worsening of DR or DME [**20**]. The discrepancy between different studies is explained by the contribution of various systemic and local, physiological, and pathological factors possibly affecting the CT.

The limitations of our study were the small sample groups, the absence of a group with DME and assessing the choroidal parameters only on a horizontal cross-sectional scan centered on the fovea. 

## Conclusion

The definition of the posterior boundary of the choroid and the segmentation method can result in differences in CT measurements. The patients with DM and those with DM and DR have altered CT and choroidal morphology, findings which need further investigation in larger sample groups that could add insight on the role of the choroid in the pathogenesis of DM and DR. 

**Conflict of Interest statement**

The authors state no conflict of interest.

**Informed Consent and Human and Animal Rights statement**

Informed consent has been obtained from all individuals included in this study.

**Authorization for the use of human subjects**

Ethical approval: The research related to human use complies with all the relevant national regulations, institutional policies, is in accordance with the tenets of the Helsinki Declaration, and has been approved by the Ethics Committee of “Gr. T. Popa” University of Medicine and Pharmacy, Iași.

**Acknowledgements**

None.

**Sources of Funding**

None.

**Disclosures**

None.
